# Patients with Obesity Undergoing Roux-En-Y Gastric Bypass Versus Fundoplication for Refractory GERD: A Systematic Review and Meta-Analysis

**DOI:** 10.1007/s11695-026-08552-1

**Published:** 2026-03-16

**Authors:** Giovanna Macanhã Scremin, Pedro Bicudo Bregion, Victor Kenzo Ivano, Pandora Eloá Oliveira Fonseca, Everton Cazzo

**Affiliations:** 1 Pontifical Catholic University of Paraná, Curitiba, Brazil; 2 State University of Campinas, Campinas, Brazil; 3 Federal University of Campina Grande, Cajazeiras, Brazil

**Keywords:** Gastric bypass, Fundoplication, GERD, Obesity, Bariatric surgery

## Abstract

**Background:**

Gastroesophageal reflux disease (GERD) significantly impairs quality of life and is associated with complications such as Barrett’s esophagus and esophageal adenocarcinoma. Obesity exacerbates GERD pathophysiology by elevating intra-abdominal pressure, making treatment more difficult. Current evidence suggests that Roux-en-Y gastric bypass (RYGB) offers superior outcomes compared to fundoplication in patients with severe obesity (BMI ≥ 40 kg/m²). This review aims to critically evaluate fundoplication versus RYGB in the population with obesity and GERD.

**Methods:**

We conducted a systematic review and meta-analysis in accordance with PRISMA guidelines. We performed a comprehensive search across PubMed, Embase, and Cochrane databases for studies comparing fundoplication versus RYGB in patients with obesity and GERD. Data extraction was standardized, focusing on intraoperative complications, operative time, length of hospital stay, reoperation, postoperative complications, postoperative dysphagia, and DeMeester score. Statistical analysis was performed using Cochrane RevMan (Review Manager 9.7.1), employing random-effects models. Heterogeneity was assessed using Cochran’s Q test and I² statistic.

**Results:**

The analysis included 7 observational studies. We found no differences in complication rates after sensitivity analysis. There were no differences in dysphagia, reoperation rate, operative time and length of stay. At a weighted mean follow-up of 42.3 months (range: 19.6 to 52.0) for fundoplication and 35.2 months (range: 14.6 to 49.0) for RYGB, GERD resolution, measured by the DeMeester score, slightly favored fundoplication, although the absolute difference was not clinically relevant. Regarding weight outcomes, RYGB demonstrated significantly higher total weight loss (TWL) at the 12-month follow-up.

**Conclusion:**

Fundoplication appears statistically superior for GERD resolution postoperatively, but the difference is not clinically relevant. RYGB has a higher TWL. Both procedures are safe for GERD control in patients with obesity. The choice between procedures should weigh reflux severity, complication risks, and metabolic diseases associated with obesity. Larger studies are needed to clarify the impact of surgical timing and patient-specific factors.

**Supplementary Information:**

The online version contains supplementary material available at 10.1007/s11695-026-08552-1.

## Introduction

Gastroesophageal reflux disease (GERD) is a highly prevalent condition, particularly in Western populations. Management typically involves pharmacological, behavioral, and dietary measures, with weight reduction representing a central component of treatment [1]. In individuals with obesity, however, GERD presents unique challenges due to factors such as increased intra-abdominal pressure, higher prevalence of hiatal hernia, extrinsic gastric compression by visceral adiposity, esophageal dysmotility, and hormonal influences from peripheral estrogen conversion [2].

In patients with refractory disease despite optimal medical therapy, surgical intervention with fundoplication has traditionally been considered the procedure of choice. Nevertheless, the treatment of GERD in patients with obesity remains complex. A recent systematic review and meta-analysis demonstrated higher recurrence rates of reflux in individuals with obesity undergoing fundoplication [3]. However, no significant differences were observed regarding perioperative complications, reinterventions, or return to daily activities. For patients with severe obesity (BMI > 40), Roux-en-Y gastric bypass (RYGB) is considered the gold-standard surgical technique, as it diverts the majority of gastric acid production away from the esophagus, thereby offering superior reflux control. Furthermore, in cases of failed fundoplication, some authors advocate RYGB as a preferable alternative to redo-fundoplication, given its more definitive reflux resolution and comparable morbidity [4].

When RYGB is contraindicated, novel approaches such as the Nissen-sleeve have been proposed, showing superior reflux control compared with sleeve gastrectomy alone [5]. However, in patients with moderate obesity (BMI 30–35 or up to 40), evidence remains conflicting as to whether fundoplication alone provides adequate long-term reflux control or whether RYGB offers superior outcomes.

To address this knowledge gap, further comprehensive evidence is required to directly compare RYGB and fundoplication as primary interventions for patients with obesity with GERD, particularly in terms of safety and efficacy. Therefore, we conducted a systematic review and meta-analysis to synthesize available data and provide robust evidence to guide clinical decision-making in this challenging patient population.

## Methods

This analysis did not require ethical approval as it did not involve any human or animal subjects. The review has been registered with the National Institute for Health Research International Registry of Systematic Reviews (PROSPERO, XXXXXXXX).

### Search Strategy

We performed a comprehensive literature search on PubMed, Cochrane Library, and Embase databases, from their inception to August 2025, to identify contemporary studies reporting short and long-term outcomes between primary fundoplication and RYGB in patients with obesity. We used the following search strategy in the databases: (“Roux-en-Y gastric bypass” OR “Roux” OR “RYGB” OR “Gastric Bypass” OR “bariatric surgery”) AND (fundoplication OR “Nissen” OR “Toupet” OR “Lind” OR “Dor”). We manually reviewed the reference lists of previous meta-analyses and employed the snowballing technique to enhance our reference list.

### Study Selection

The study selection was conducted in accordance with the Preferred Reporting Items for Systematic Reviews and Meta-Analyses (PRISMA) guidelines. Following the removal of duplicate records, two independent reviewers (GS and VK) conducted the initial screening. A third reviewer (EC) was consulted to resolve any conflicts. Titles and abstracts were assessed using predefined inclusion and exclusion criteria, and full-text versions of articles considered relevant were obtained for further evaluation.

To ensure a comprehensive overview of the surgical management of GERD in patients with obesity, we included studies regardless of specific phenotypic presentations of the disease. Specifically, studies involving patients with large paraesophageal hernias (Ducoin et al.) and those focusing on patients with Barrett’s esophagus (Braghetto et al.) were eligible for inclusion, as these conditions represent common clinical scenarios within the spectrum of refractory GERD in this population.

Data sources varied among the included studies, ranging from single-center cohorts to large-scale administrative databases. Notably, the study by Varela et al. utilized a national administrative database, which allowed for a significantly larger sample size regarding early postoperative complications, although it lacks long-term functional outcomes.

To address potential clinical heterogeneity, we performed sensitivity analyses by excluding studies with very specific populations (large hernias and Barrett’s esophagus) and those based on administrative data to verify if the primary outcomes remained consistent across the ‘typical’ GERD population.

### Eligibility Criteria

Articles eligible for this review were observational studies or randomized trials written in English comparing weight loss outcomes, GERD resolution, and complications in adult patients (18–70 years old) with obesity (BMI > 30 kg/m²) who underwent primary fundoplication and RYGB.

The exclusion criteria included non-English publications, studies without relevant outcomes for the review, conference abstracts and proceedings, case reports, non-comparative study designs, and studies that analyzed revisional fundoplication or revisional RYGB. Table 1 of the Supplementary Material shows the quality of included studies using the ROBINS-I v2.

**Table 1:** preoperative characteristics in fundoplication and RYGB patients

Author	Year	*N* FP	*N* RYGB	Age FP (years)	Age RYGB (years)	BMI FP (kg/m²)	BMI RYGB (kg/m²)	Hiatal Hernia (%) FP/RYGB
Betzler et al.	2018 to 2021	55	38	65 ± 17	51 ± 12	28.24 ± 17.28	32.81 ± 11.78	87.3/52.6
Braghetto et al.	NR	79	21	41.3	53.2	33.9 ± 2.9	43.7 ± 4.2	NR
Colvin et al.	2015 to 2019	24	20	51.5 ± 15.4	42.8 ± 11.4	39.2 ± 1.49	44.21 ± 8.45	92/80
Ducoin et al.	2013 to 2021	40	16	64.0 ± 10.8	48.6 ± 12.2	34.2 ± 3.3	39.2 ± 6.2	100/100
Joseph et al.	2009 to 2021	72	23	63 ± 12	56 ± 9	NR	NR	NR
Patterson et al.	1995 to 2000	6	6	50.8	39.8	39.8	55	NR
Varella et al.	2004 to 2007	6,108	21,156	NR	NR	NR	NR	NR

*N* number, *FP* fundoplication, *RYGB* Roux-en-Y gastric bypass, *BMI* body mass index, *NR* not reported

Data extraction was carried out and reviewed by two independent authors (GS and VK) using Excel spreadsheets, with a third author (PB) verifying accuracy. The extracted variables included study characteristics (publication year, sample size on each group, study design, maximum follow-up, and reported comorbidities) as well as patient demographics (age, sex, and body mass index – BMI).

### Outcomes

Primary outcomes were GERD resolution and complication rates. GERD resolution was defined as a composite outcome including objective normalization (postoperative DeMeester score < 14.1) and subjective clinical improvement as measured by validated questionnaires (e.g., GERD-HRQL, RSI, GERSS). Secondary outcomes were weight loss, operation characteristics (reoperation, length of stay, and operative time). For complication rates, we pooled intraoperative complications, postoperative complications, and dysphagia. Weight loss was assessed using the percentage of total weight loss (%TWL).

### Statistical Analysis

We conducted meta-analyses to compare the outcomes of primary fundoplication and RYGB in patients with obesity, summarizing data from registry studies. Risk Ratio (RR) with 95% confidence intervals (CI) and mean differences (MD) with 95% CIs were used to assess primary and secondary outcomes. Heterogeneity among the studies was evaluated using the Cochran Q statistic and the I² statistic. Significant heterogeneity was identified with a p-value of less than 0.10 and an I² of 50% or higher. To account for inherent clinical heterogeneity, a random-effects model was employed. All statistical analyses were performed using Review Manager software. We perform a dual-track analysis (including and excluding Varela et al.), which serves as a robustness check. This approach allows the study to benefit from the high statistical power of administrative data while verifying findings against granular clinical studies to ensure accuracy in physiological outcomes.

### Risk of Bias Assessment

The risk of bias for non-randomized studies was assessed independently by two reviewers using the Risk of Bias in Non-Randomized Studies of Interventions (ROBINS-I) tool, as recommended. The following seven domains were evaluated: (1) bias due to confounding, (2) bias in selection of participants into the study, (3) bias in classification of interventions, (4) bias due to deviations from intended interventions, (5) bias due to missing data, (6) bias in measurement of outcomes, and (7) bias in selection of the reported result. Each domain was judged as “low,” “moderate,” “serious,” or “critical”, and an overall risk of bias judgment was assigned to each study, following the ROBINS-I guidance. Disagreements were resolved through discussion, and consensus was reached for all decisions.

## Results

### Study Characteristics

**Fig. 1:**
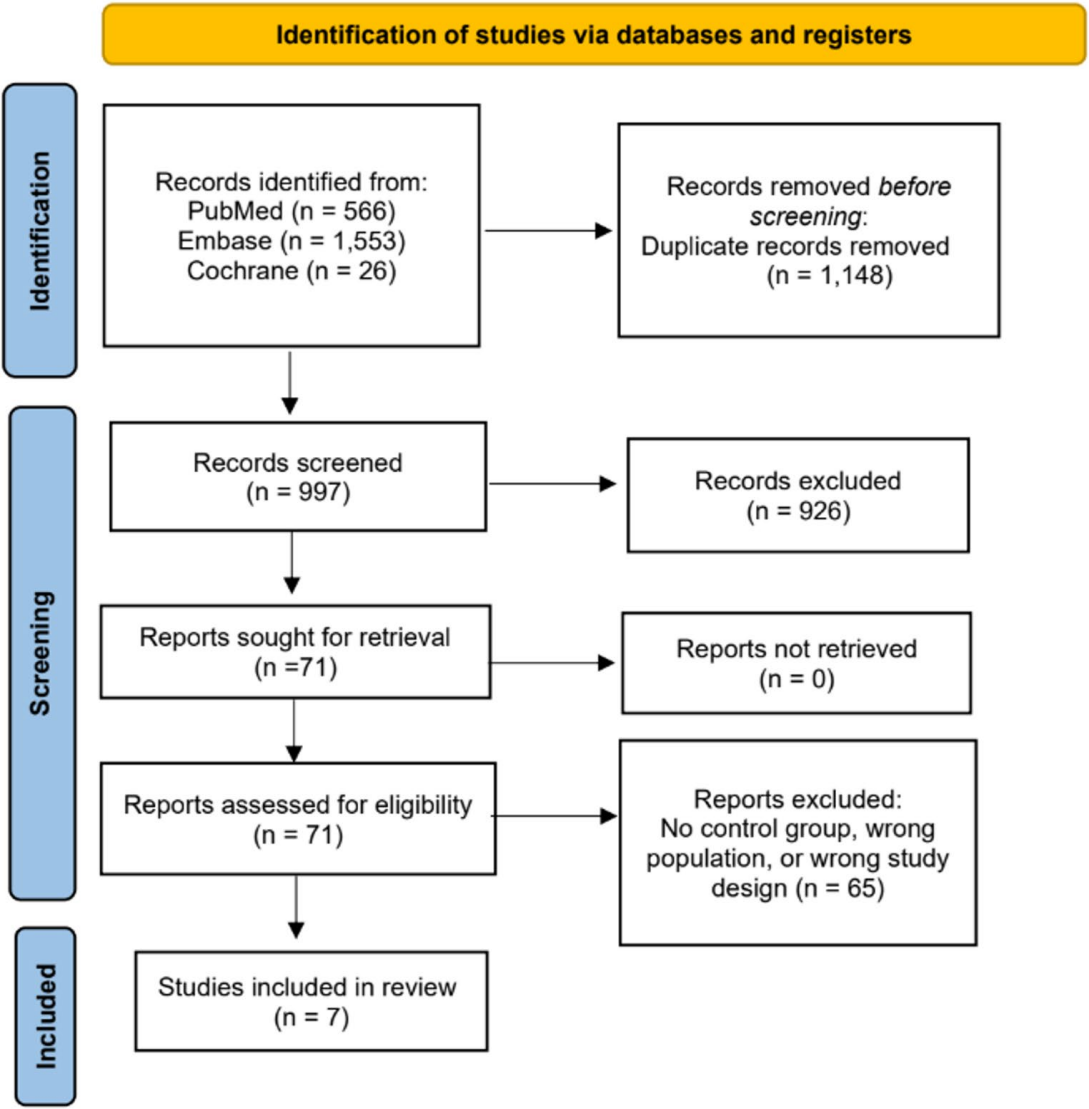
PRISMA flowchart of study selection

From the systematic search, we retrieved 997 studies, out of which 7 met the inclusion criteria for the final analysis. Figure 1 presents the PRISMA flowchart detailing the study selection process. Table 1 shows the details of the included studies published between 1995 and 2021. All 7 studies were observational comparative studies.

### Patient Characteristics

Table 1 provides a summary of the demographic characteristics of the patient populations across the studies. Across the included studies, six studies evaluated a total of 439 patients, with 276 undergoing fundoplication and 124 receiving Roux-en-Y gastric bypass (RYGB); 39 patients from Braghetto et al. were excluded from the analysis because they underwent a hybrid procedure that combined fundoplication with a distal gastrectomy and Roux-en-Y gastric bypass (FVDGRYGJ) for the treatment of long-segment Barrett’s esophagus (LSBE). The seventh study, Varella et al., used data from the University HealthSystem Consortium database using “International Classification of Diseases, 9th Revision” procedural and diagnosis codes for individuals with morbid obesity with GERD, evaluating a total of 27.264 patients’ medical records. Notably, the study by Varela et al. only examined early postoperative results and did not evaluate weight loss or the control of GERD.

The mean age of the entire cohort was 54.2 years, with fundoplication patients generally older compared to those treated with RYGB. The weighted average preoperative BMI was 35.6 kg/m², highlighting the predominance of patients with obesity, particularly in the RYGB cohorts, where BMI consistently exceeded 40 kg/m² in most studies. Women comprised most of the population, with an overall female prevalence of 75.7%, and proportions above 85% in several RYGB groups.

### DeMeester Score

**Fig. 2:**
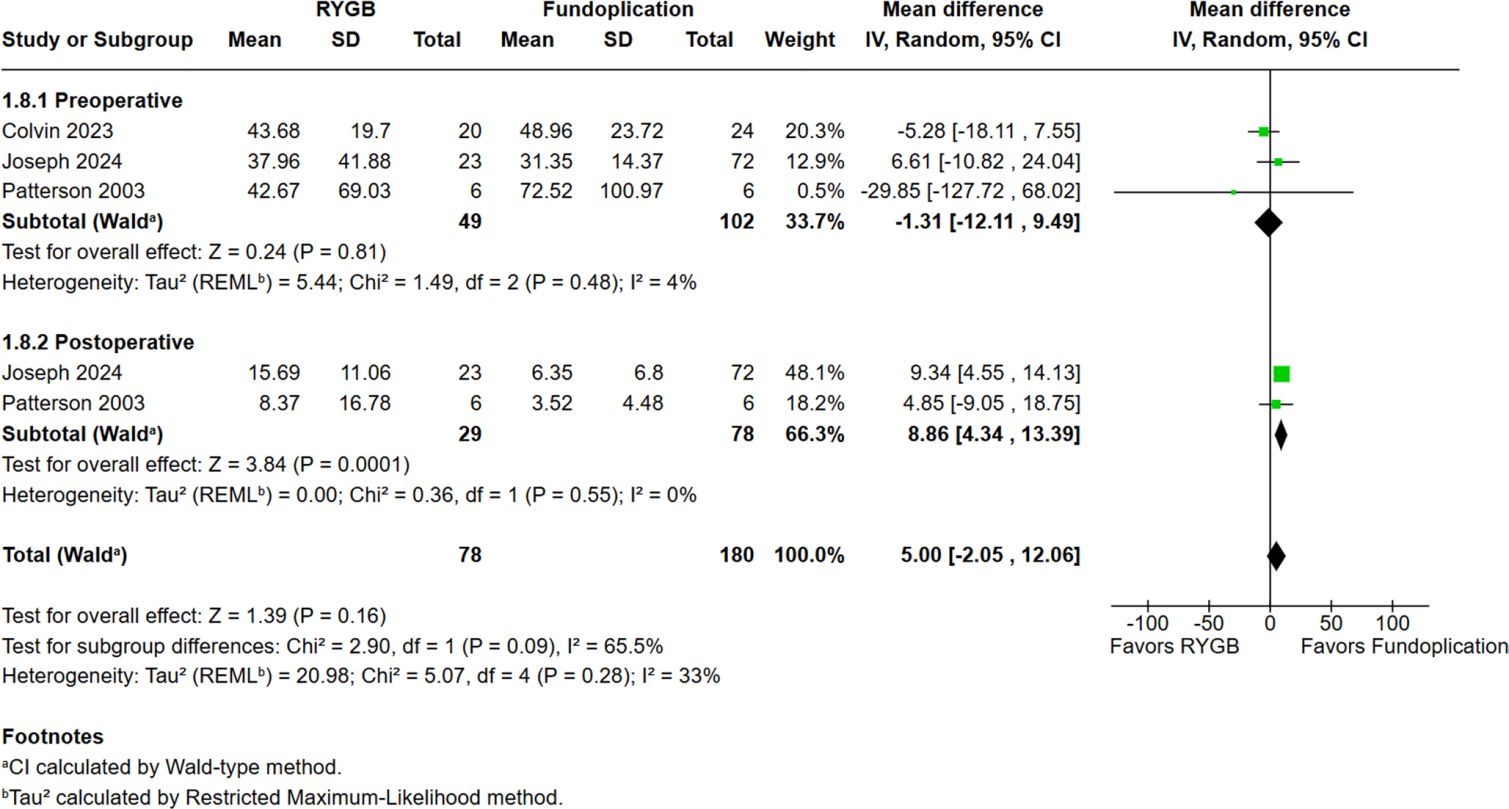
DeMeester score before and after surgery

We pooled preoperative and postoperative data, comparing a total of 78 patients. Figure 2 shows there was no difference between fundoplication and RYGB (MD = −1.31; 95% CI −12.11 to 9.49; *p* = 0.81; I²= 4%) in preoperative DeMeester score. After surgery, fundoplication shows a slightly lower score (MD = 8.86; 95% CI 4.34 to 13.39; *p* = 0.0001; I² = 0%). The follow-up duration for objective reflux assessment via the DeMeester score reached a weighted mean of 42.3 months (range: 19.6 to 52.0) for the fundoplication group and 35.2 months (range: 14.6 to 49.0) for the RYGB group.

### Complications

**Fig. 3:**
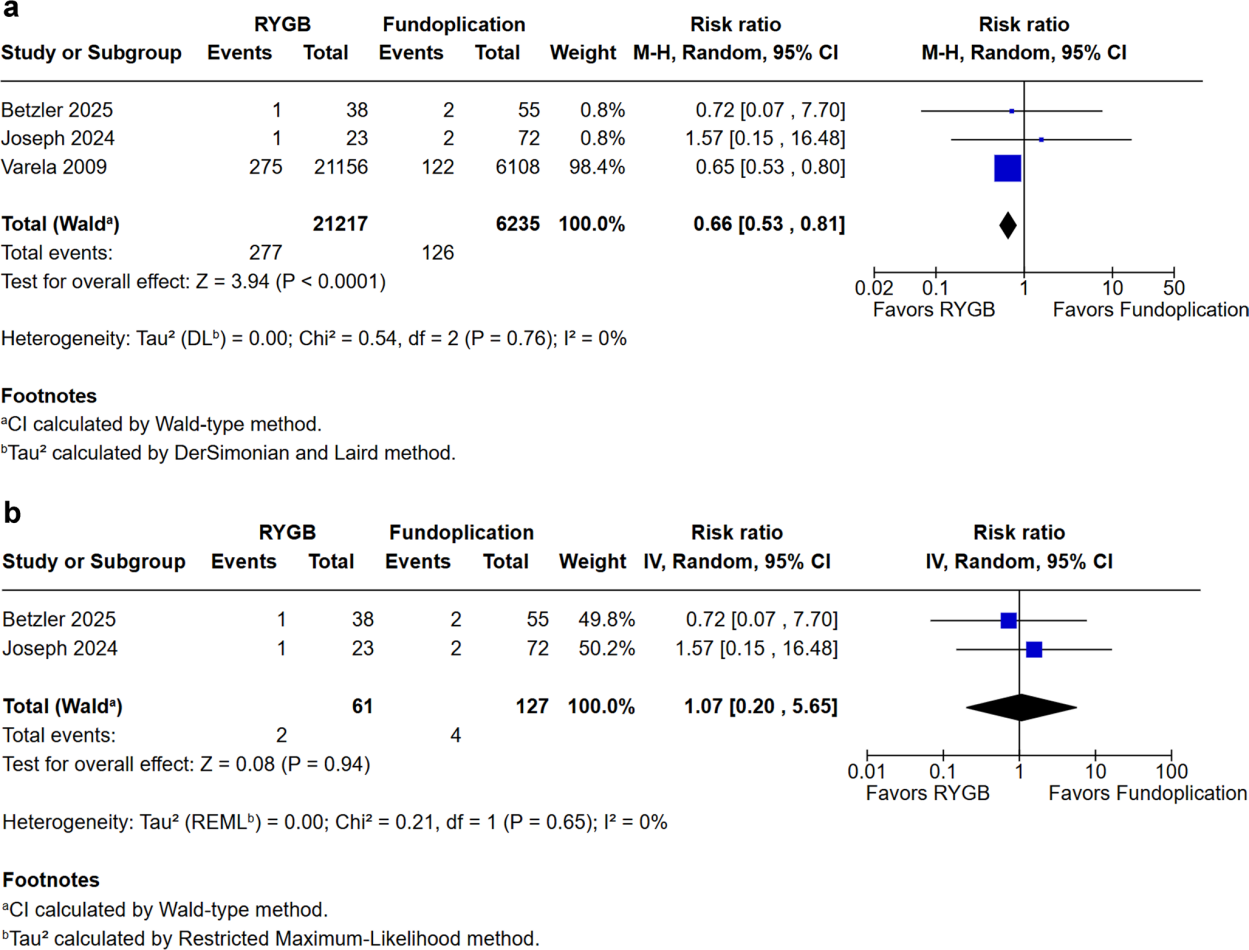
**a.** Intraoperative complications. **b.**Intraoperative complications excluding the study by Varela et al

**Fig. 4:**
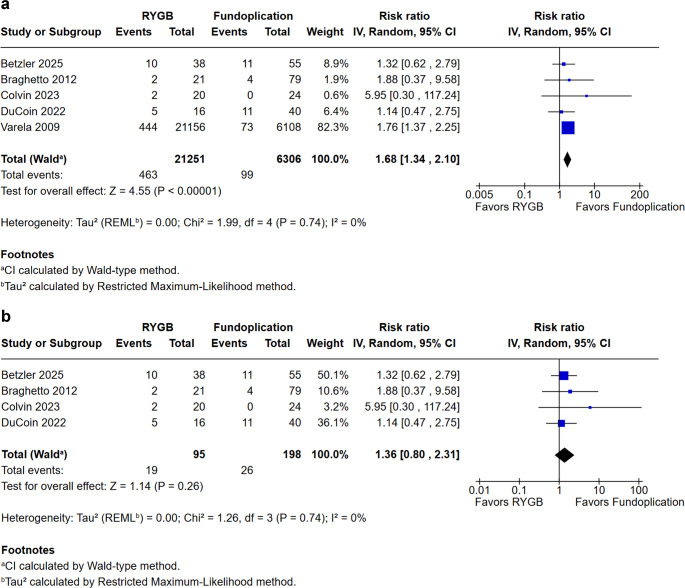
**a. **Postoperative complications. **b.**Postoperative complications in prospective studies excluding the study by Varela et al

**Fig. 5:**
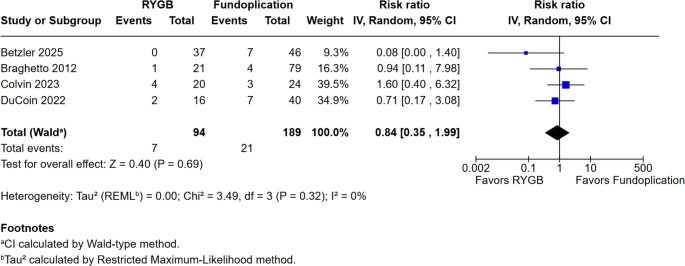
Postoperative dysphagia

In our analysis of complications, we evaluated intraoperative complications, postoperative complications, and dysphagia (Figs. 3, 4 and 5). The reporting and classification of complications varied across the included studies. Betzler et al. employed the most rigorous methodology, utilizing both the Clavien-Dindo classification and the Comprehensive Complication Index (CCI) to record events during the initial hospitalization and at the 3- and 12-month follow-ups. Similarly, DuCoin et al. used the Clavien-Dindo system to categorize both in-hospital complications, such as leaks and perforations, and post-discharge outcomes, including hernia recurrence. In contrast, Varela et al. relied on an administrative model from the UHC database, which uses a complication profiler and comorbidity software to assign disease severity levels (minor, moderate, or major) and identify specific adverse events prior to discharge. The remaining studies (Braghetto et al., Colvin et al., Joseph et al., and Patterson et al.) did not utilize universal scoring systems like Clavien-Dindo, instead basing their findings on direct clinical reports, reoperation rates, hospital readmissions, and observed operative morbidity.

Betzler et al. defined preoperative complications as GERD-related risks like aspiration and esophagitis, while postoperative events included intraoperative injuries (pleural/esophageal) and follow-up issues such as dysphagia, leaks, and ulcers. Braghetto et al. viewed preoperative complications as esophageal ulcers or strictures resulting from Barrett’s, with postoperative outcomes focusing on dumping syndrome, diarrhea, and surgical leaks. Colvin et al. highlighted Barrett’s esophagus and cancer as preoperative concerns, whereas postoperative complications involved marginal ulcers, bloating, and the need for dilation. Joseph et al. considered esophagitis and adenocarcinoma as preoperative complications, noting a “complicated hospital course” involving respiratory issues postoperatively. Ducoin et al. described preoperative complications through chronic respiratory symptoms (cough/dyspnea), while postoperative events included hernia recurrence, intestinal obstructions, and arrhythmias. In contrast, Patterson et al. noted no postoperative complications in their small sample, despite preoperative comorbidities like sleep apnea. Finally, Varela et al. used a broad administrative definition, categorizing postoperative complications into procedure-related (e.g., perforation, bleeding) and non-procedure-related (e.g., pneumonia, thromboembolism) based on illness severity.

Intraoperative complication rates (Fig. 3) demonstrated a significant reduction in patients undergoing RYGB compared with fundoplication (RR = 0.66; 95% CI 0.53 to 0.81; *p* < 0.0001; I² = 0%). This finding indicates greater intraoperative safety associated with the bariatric approach. Conversely, postoperative complications (Fig. 4) were more frequent in the RYGB group (RR = 1.68; 95% CI 1.34 to 2.10; *p* < 0.00001; I² = 0%).

Importantly, the study by Varella et al. accounted for 98.4% and 82.3% of the total complications, and due to its database design, we conducted a sensitivity analysis excluding this trial. After removal, no statistically significant difference was observed between groups, suggesting that the initial finding was predominantly driven by this large database (Figs. 3a and 4a).

Finally, dysphagia outcomes are presented in Fig. 5, where pooled results highlighted no consistent differences between procedures (RR = 0.84; 95% CI 0.35 to 1.99; *p* = 0.69; I² = 0%). Across the analyzed studies, the definition and assessment of dysphagia varied significantly. Colvin et al. utilized a dual approach, defining clinically significant dysphagia as that requiring endoscopic dilation, while also tracking symptom severity via GERSS. Betzler et al. assessed the condition through the ‘Eating/Drinking Problems’ domain of the QOLRAD questionnaire, whereas Joseph et al. relied on specific patient-reported dysphagia scores. In contrast, DuCoin et al. and Braghetto et al. evaluated dysphagia as a binary variable based on clinical reports. Lastly, Varela et al. and Patterson et al. did not provide a formal definition for this symptom, focusing on major complications or objective reflux scores.

### Operative Characteristics Outcomes

**Fig. 6:**
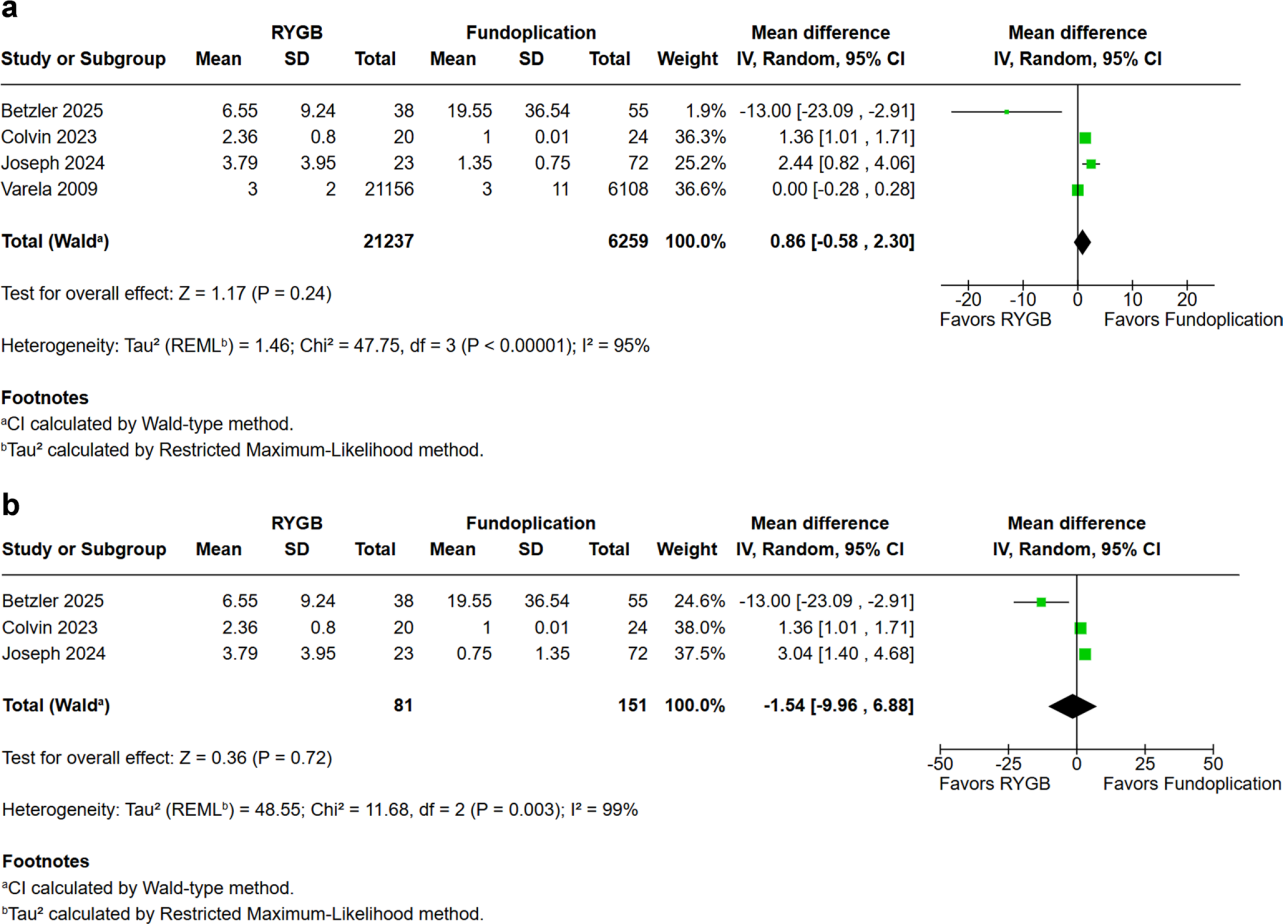
**a**. Length of stay in days **b**.Length of stay in days in prospective studies excluding the study by Varela et al

**Fig. 7:**
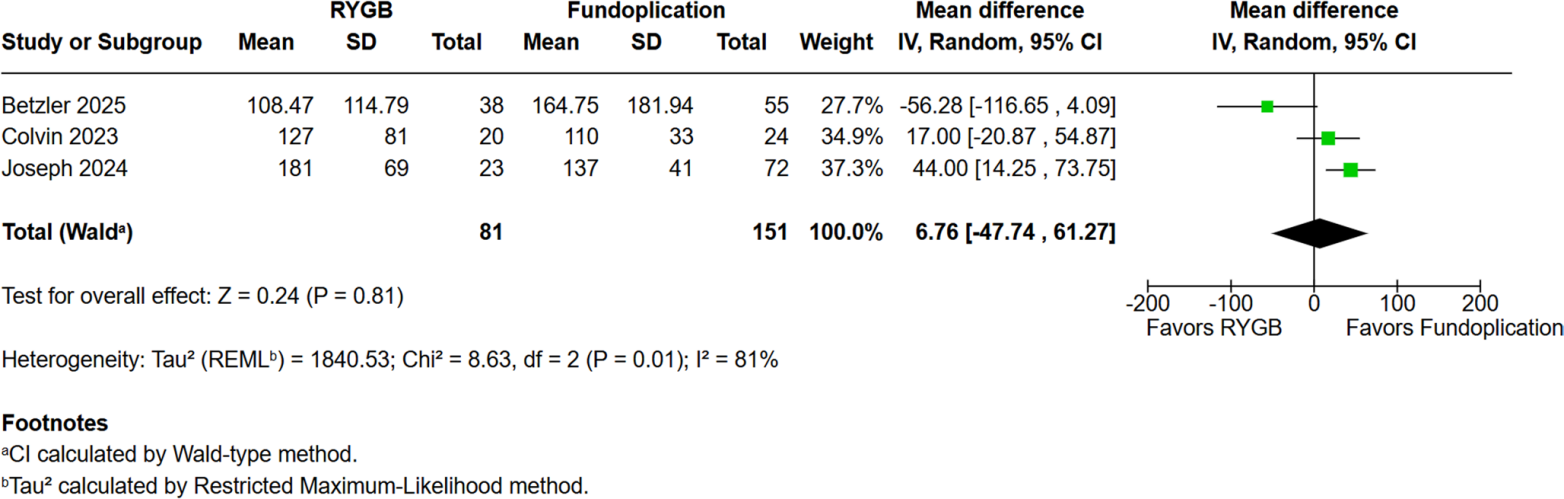
Operative time in minutes

**Fig. 8:**
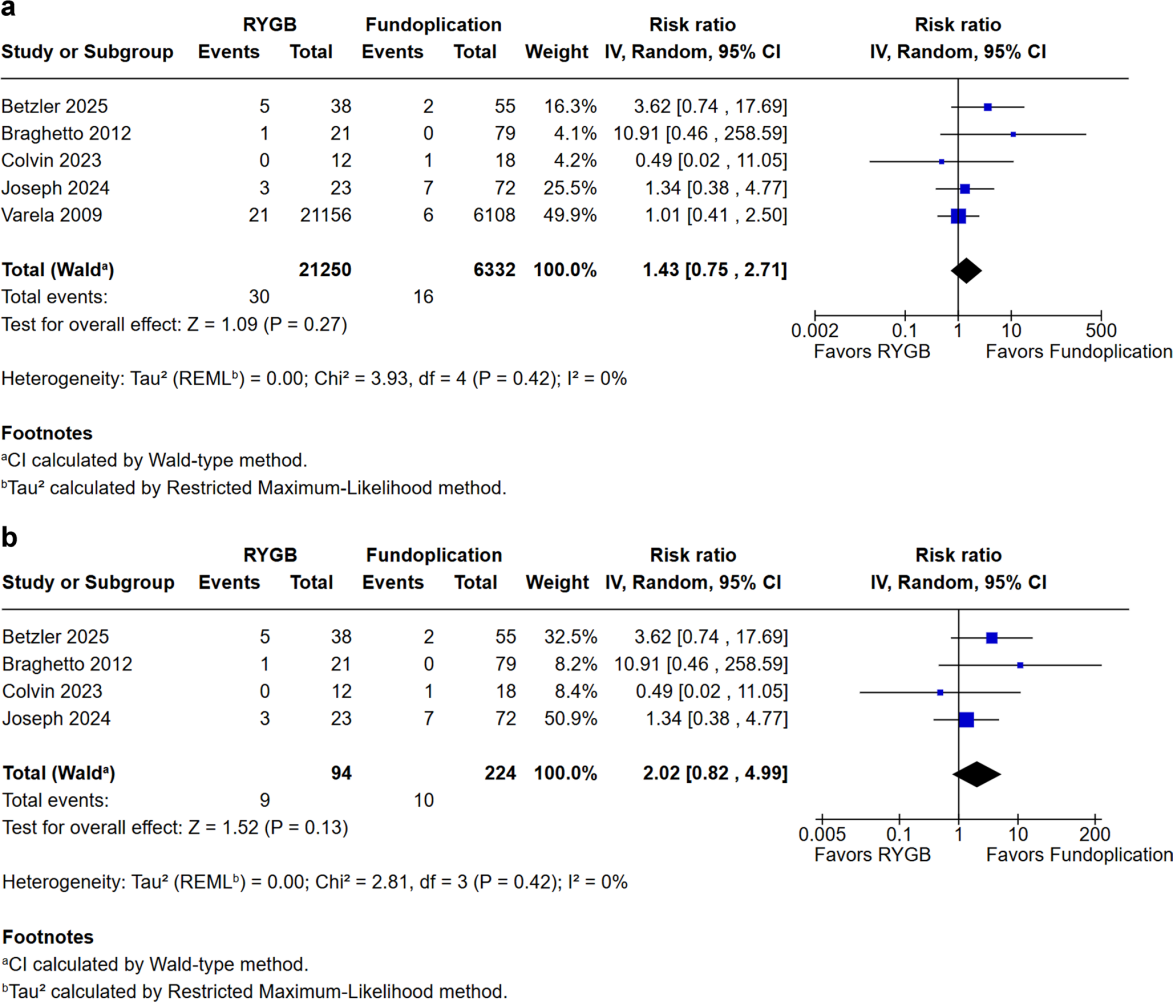
**a**. Reoperation rates **b**. Reoperation rates excluding the study by Varela et al

We conducted a pooled analysis of length of stay (LOS), operative time, and reoperation rates to compare operative characteristics between RYGB and fundoplication (Figs. 6, 7 and 8). No statistically significant difference was observed in LOS between the two groups (MD = 0.86 days; 95% CI − 0.58 to 2.30; *p* = 0.24; I² = 95%). After performing a sensitivity analysis by removing the Varela et al. study (Fig. 6A), the results remained stable (RR = −1.54; 95% CI −9.96 to 6.88; *p* = 0.72; I² = 99%). Similarly, operative time did not differ significantly (MD = 6.76 min; 95% CI − 47.74 to 61.27; *p* = 0.81; I² = 81%).

Reoperation rates were slightly higher for the RYGB group in the overall analysis, also (RR = 1.43; 95% CI 0.75 to 2.71; *p* = 0.27; I² = 0%; Fig. 8). This trend persisted in the sensitivity analysis excluding Varela et al. (Fig. 8A), although the difference was statistically no longer significant (RR = 2.02; 95% CI 0.82 to 4.99; *p* = 0.13; I² = 0%).

### %TWL

**Fig. 9:**
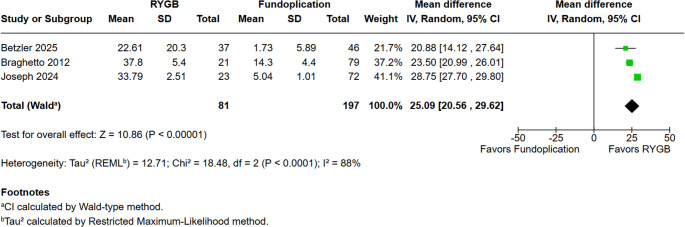
Total weight loss at 12 months

To assess weight loss, we evaluated the percentage of total weight loss (%TWL) at follow-up at 12 months post-surgery. RYGB was associated with significantly greater %TWL compared with fundoplication (MD = 25.09; 95% CI 20.56 to 29.62; *p* < 0.00001), although substantial heterogeneity was observed (I² = 88%) (Fig. 9).

### Patient-Reported Outcomes

Across studies, both fundoplication and RYGB significantly improved reflux-related symptoms; however, the durability and magnitude of the effects varied. In the propensity-matched analysis by Betzler et al., reflux symptom regression at 3 months was significantly higher after RYGB compared with fundoplication (92.3% vs. 25%; *p* = 0.0223), although the difference was not statistically significant at 1 year. Importantly, RYGB patients reported superior postoperative quality of life as measured by the BQL and QOLRAD sleep domains (*p* = 0.0183). Similarly, Colvin et al. demonstrated significant improvement in GERD-HRQL and GERSS scores after both laparoscopic Nissen fundoplication (LNF) and RYGB. GERD-HRQL decreased from 29 to 1.5 in the LNF group and from 36 to 9 in the RYGB group (both *p* < 0.01).

GERSS scores showed parallel improvements, with a trend toward lower mid-term scores after LNF, although this was not statistically significant. Conversely, Joseph et al. observed more durable symptom control at follow-up: at 2 and 5 years, GERD-HRQL scores were significantly better after fundoplication compared to RYGB (4.9 vs. 10.2, *p* = 0.045; 3.3 vs. 9.0, *p* = 0.041), suggesting potential superiority of fundoplication for selected patients. Patterson et al. confirmed that both procedures effectively reduced heartburn, with no significant difference postoperatively. However, preoperative symptom severity was greater in the fundoplication group, which showed greater relative improvement.

### Esophageal Physiology and Manometry

Manometric findings varied across studies. Braghetto et al. had 3 groups, to maintain clinical homogeneity and strictly compare standalone procedures, patients from the Braghetto et al. study who underwent the hybrid FVDGRYGJ procedure were excluded from this analysis. This specific group combined fundoplication with distal gastrectomy, which does not align with the primary objective of comparing primary fundoplication versus standard RYGB. Despite this, abnormal acid reflux remained uncommon after RYGB (9.5%), compared with 24% after fundoplication in hypotensive LES patients.

In contrast, Patterson et al. observed normalization of LES resting pressures after both LNF and RYGB. Mean LES improved from 12.9 to 35.5 mmHg after LNF (*p* < 0.003), and from 23.6 to 29.7 mmHg after LGB, though the latter was not statistically significant. Both groups achieved postoperative LES pressures within the normal range. However, it is important to note that this specific analysis included only 12 patients. While providing objective data, the small sample size in this subset limits the statistical power to draw definitive conclusions regarding manometric changes.

### Barrett’s Esophagus and Endoscopic Outcomes

Regarding Barrett’s esophagus, Braghetto et al. documented that metaplasia regressed in 51.9% in the fundoplication group and in 61.9% in the RYGB group, with no progression to dysplasia observed in either group. These findings support both fundoplication and RYGB as effective interventions for controlling disease progression, though mechanisms differ (anatomical reinforcement vs. acid diversion).

### Risk of Bias

The risk of bias assessment of the included non-randomized studies, conducted using the ROBINS-I tool, revealed that most of the available evidence presents significant methodological limitations, resulting in an overall high risk of bias. Specifically, five out of seven studies were rated as having a critical risk of bias (Betzler et al [6], Colvin et al [7], DuCoin et al. [8], Joseph et al. [9], Patterson et al. [10]), one as serious (Braghetto et al. [11]), and only one as moderate (Varela et al. [12]). The domains most frequently associated with elevated risk were due to confounding, outcome measurement, and missing data.

## Discussion

This systematic review and meta-analysis included seven observational comparative studies, evaluating the efficacy and safety of fundoplication versus RYGB for the management of GERD, a condition frequently associated with obesity. Both procedures were effective in improving reflux symptoms and disease burden. Patients undergoing RYGB typically had higher BMI and more metabolic comorbidities, whereas those undergoing fundoplication were generally older and had more cardiovascular and pulmonary conditions. Postoperatively, both interventions significantly reduced GERD scores, but RYGB produced superior weight loss at 12 months.

It is important to acknowledge that some of the included studies focused on specific clinical phenotypes within the spectrum of GERD and obesity, which contributes to the observed clinical heterogeneity. Specifically, DuCoin et al. evaluated patients who, in addition to obesity and GERD, presented with large paraesophageal hernias, a condition that inherently increases surgical complexity and may influence recurrence rates. Similarly, Braghetto et al. focused their analysis on patients with Barrett’s esophagus, representing a subgroup with more advanced mucosal damage and potentially different physiological responses to anti-reflux procedures. While these studies provide valuable data on complex cases, their unique population characteristics must be considered when generalizing the findings to the typical patient with uncomplicated refractory GERD and obesity.

The total number of patients included in this meta-analysis was predominantly influenced by the study by Varela et al., which contributed 27,264 cases derived from a national administrative database. This large-scale cohort provides significant statistical power for evaluating early postoperative complications and hospital resource utilization. In contrast, the remaining six studies (Betzler, Braghetto, Colvin, Ducoin, Joseph, and Patterson) provided a combined total of 439 patients.

These smaller clinical cohorts differ significantly from the administrative data as they offer granular, patient-level clinical outcomes, including objective reflux measurements (DeMeester scores), manometric data, and long-term follow-up that are not available in large databases. To ensure that the overwhelming sample size of the Varela study did not skew our clinical conclusions, we performed sensitivity analyses for all primary outcomes, comparing the results with and without the administrative data. This approach allowed us to balance the broad epidemiological insights of ‘big data’ with the precise clinical evidence required for surgical decision-making in GERD.

Intraoperative complications were lower with RYGB, while postoperative complications were initially higher, though this difference was no longer significant after sensitivity analysis excluding the large Varella et al. cohort. No consistent differences were observed in dysphagia, length of stay, operative time, or reoperation rates. Patient-reported outcomes generally favored RYGB in the short term, while some long-term data suggested more durable reflux control with fundoplication. Both procedures improved esophageal physiology, and RYGB prevented recurrent acid reflux.

Comparative outcomes revealed important distinctions. Fundoplication achieved a modest but statistically significant reduction in DeMeester scores. At the same time, RYGB demonstrated greater short-term improvements in reflux symptoms and quality of life (Betzler et al.). In contrast, long-term follow-up showed more sustained GERD control with fundoplication (Joseph et al.). RYGB was associated with fewer intraoperative complications but higher postoperative adverse events, largely driven by Varella et al. Both procedures achieved comparable improvements in dysphagia, reoperation rates, and hospital stay. Manometric data were inconsistent: Braghetto et al. reported increased LES pressure after fundoplication, while Patterson et al. observed normalization after both procedures. Regarding Barrett’s esophagus, Braghetto et al. found regression of intestinal metaplasia after either operation, supporting their role in disease modification.

Recent guidelines and studies provide additional context. The 2022 ACG Clinical Guideline highlights that obesity complicates fundoplication, increasing technical difficulty, recurrence, and postoperative risks, whereas RYGB is considered the preferred antireflux operation in individuals with obesity, addressing both GERD and obesity. Nonetheless, some studies reported no significant differences in perioperative outcomes between patients either with or without obesity undergoing fundoplication, though recurrence was higher in patients with obesity [13].

A retrospective cohort of patients with class I–II obesity and refractory GERD showed that, after propensity-score matching, RYGB resulted in shorter operative time and hospital stay, lower complication and dysphagia rates, and greater reflux improvement at three months, despite higher baseline BMI and comorbidities [6]. Another analysis reported that laparoscopic Nissen fundoplication (LNF) and RYGB provided comparable improvements in disease-specific quality of life, symptom control, and satisfaction at mid-term follow-up, with no significant differences in complications, adverse effects, or reoperations [7].

Evidence from paraesophageal hernia repair in patients with obesity indicates that fundoplication and gastric bypass achieve similar reflux resolution, but gastric bypass results in lower hernia recurrence and greater weight loss [8]. For failed fundoplication, conversion to RYGB provides superior reflux control compared with redo fundoplication, particularly in individuals with obesity, though at the expense of higher perioperative complication risk in complex cases [14, 15]. A recent narrative review concluded that while fundoplication remains the gold standard for the general population, its efficacy is uncertain in severe obesity, where RYGB is often favored due to its dual benefit on reflux and weight management [16].

Unlike prior reviews, our analysis pooled a larger sample and performed sensitivity analyses, revealing no consistent differences in dysphagia, length of stay, operative time, or reoperations, contrasting with some reports suggesting higher dysphagia rates after fundoplication [17].

Overall, current evidence suggests that RYGB is generally the preferred surgical approach for patients with obesity and GERD, particularly those with higher BMI, refractory disease, or failed fundoplication, given its similar GERD control and metabolic advantages. Nevertheless, carefully selected patients may achieve comparable mid-term outcomes with either procedure [6–8, 13–16, 18–20].

### Limitations

This study has several limitations that warrant consideration. First, most of the included data originated from non-randomized, retrospective cohorts, which inherently limits causal inference. We observed considerable heterogeneity regarding surgical techniques, follow-up durations, and the definition of outcomes across the trials. Furthermore, the total patient count was heavily skewed by a single large-scale administrative database, and while sensitivity analyses were performed to mitigate this, conclusions regarding physiological outcomes—such as lower esophageal sphincter (LES) pressure—were based on a very small subset of patients (Patterson et al., *n* = 12), which lacks sufficient statistical power.

Selection bias was also evident, as RYGB patients generally presented with higher BMIs and more metabolic comorbidities, whereas fundoplication was often preferred for older patients with more cardiopulmonary diseases. Finally, the inclusion of specific populations, such as those with large paraesophageal hernias or Barrett’s esophagus, adds clinical complexity that may not represent the ‘typical’ GERD patient. Consequently, the overall certainty of the evidence remains low, and the results should be interpreted with caution.

## Conclusion

Although both fundoplication and RYGB effectively alleviate GERD symptoms and improve patient-reported outcomes, RYGB demonstrates superior weight loss, metabolic benefits, and potential for lower recurrence of hiatal hernia and dysphagia. Fundoplication, remains a viable option for individuals with obesity who are not suitable candidates for gastric bypass, offering good symptom control with a relatively low complication rate. Overall, the consensus among included studies favors RYGB as the procedure of choice for patients with obesity and refractory GERD, particularly when metabolic comorbidities coexist. Nevertheless, given the predominance of non-randomized data and the high risk of bias, further prospective randomized controlled trials with standardized outcome reporting are needed to establish definitive clinical recommendations.

## Supplementary Information

Below is the link to the electronic supplementary material.

Supplementary Material 1 (DOCX 558 KB)

## Data Availability

No datasets were generated or analysed during the current study.
