# Supplementary figures and images for: Patients with Obesity Undergoing Roux-En-Y Gastric Bypass Versus Fundoplication for Refractory GERD: A Systematic Review and Meta-Analysis
Source: Obes Surg. 2026 Mar 16;36(4):1909–21. doi: 10.1007/s11695-026-08552-1 (PMC13083527; doi:10.1007/s11695-026-08552-1)

**Supplementary material**

**Table 1.** Risk of bias

**
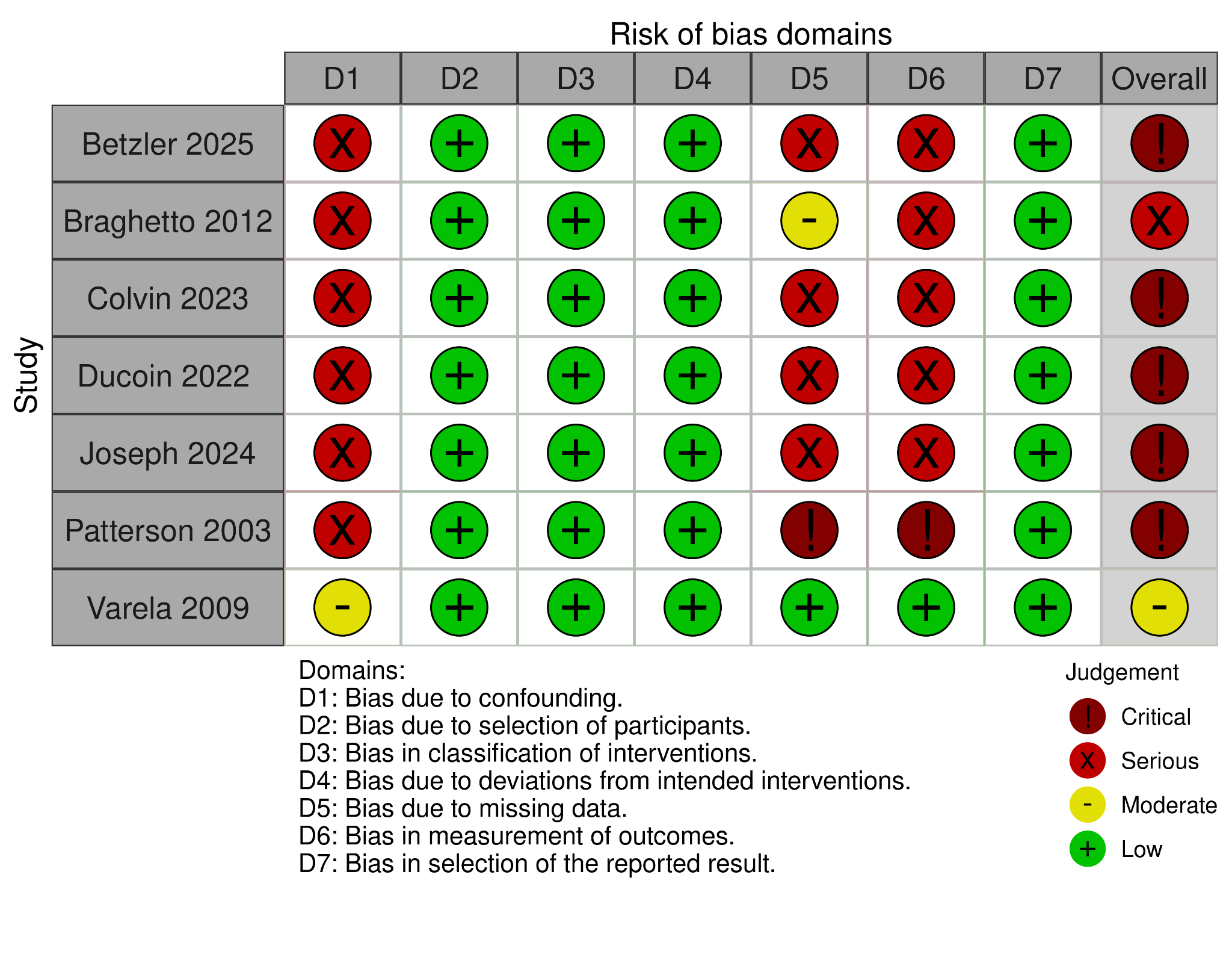
**

Supplement: Supplementary file 1 — Supplementary Material 1 (DOCX 558 KB) [file 11695_2026_8552_MOESM1_ESM.docx]
